# TM_02_ Quarter-Mode Substrate-Integrated Waveguide Resonator for Dual Detection of Chemicals

**DOI:** 10.3390/s18061964

**Published:** 2018-06-18

**Authors:** Ahmed Salim, Sungjoon Lim

**Affiliations:** School of Electrical and Electronics Engineering, College of Engineering, Chung-Ang University, 221, Heukseok-Dong, Dongjak-Gu, Seoul 156-756, Korea; ahmedsalim789@gmail.com

**Keywords:** dual detection, TM_02_-mode quarter-mode substrate-integrated waveguide, microwave sensor, microfluidic channel

## Abstract

The detection of multiple fluids using a single chip has been attracting attention recently. A TM_02_ quarter-mode substrate-integrated waveguide resonator designed at 5.81 GHz on RT/duroid 6010LM with a return loss of 13 dB and an unloaded quality factor of Q ≈ 13 generates two distinct strong electric fields that can be manipulated to simultaneously detect two chemicals. Two asymmetric channels engraved in a polydimethylsiloxane sheet are loaded with analyte to produce a unique resonance frequency in each case, regardless of the dielectric constants of the liquids. Keeping in view the nature of lossy liquids such as ethanol, the initial structure and channels are optimized to ensure a reasonable return loss even in the case of loading lossy liquids. After loading the empty channels, Q is evaluated as 43. Ethanol (E) and deionized water (DI) are simultaneously loaded to demonstrate the detection of all possible combinations: [Air, Air], [E, DI], [DI, E], [E, E], and [DI, DI]. The proposed structure is miniaturized while exhibiting a performance comparable to that of existing multichannel microwave chemical sensors.

## 1. Introduction

The monitoring of several parameters at different processing steps is common in chemical industries and pharmaceutical plants and in quality control for the food industry [1]. Multiple sensors or a sensor array can be employed in these scenarios; however, these approaches have a large footprint, a complex design, and/or expensive fabrication for mass production. A radiofrequency (RF) sensor chip realized from components and monolithic devices can be utilized as a multiple sensing device; however, its high power consumption could limit its widespread adoption. As an alternative method, simultaneous detection of multiple fluids using a single-chip sensor is the main objective of this study.

Radio frequency (RF) technology-based sensors are generally noninvasive, small, inexpensive, and easily fabricated compared with nonelectromagnetic sensors. Moreover, they are noncontact and operate in the ambient environment. However, their sensitivity (lower limit of detection) is significantly lower than that of nonelectromagnetic sensors. For instance, in [2], a microtoroid-based label-free optical resonator (which works on total internal reflection) exhibited an excellent limit of detection on the order of a single protein molecule (2.5-nm radius). In addition, RF sensors lack selectivity, which is a serious drawback from a practical viewpoint. Trends in RF sensors include enhancing the performance, miniaturization, and adding functionality, such as the capability to detect multiple chemicals. Additionally, hybrid sensors (RF sensors with additional coatings) have been utilized to obtain RF sensors with selectivity.

Rectangular waveguide resonators render electric-field (E-field) energy confined in a localized area and reduce radiation losses and effects that arise from a high resistance [3]. Substrate-integrated waveguide (SIW) technology exhibits advantages of rectangular waveguides (such as low radiation losses, low leakage losses, and high Q) and planar circuit boards (such as a planar structure, easy fabrication, small size, and lightweight structures) [4,5,6]. A strong E-field (energy) centered at the SIW cavity can be utilized for various sensing applications. A high quality-factor (Q ≈ 300) SIW-based microwave humidity sensor has been proposed [7]. To realize a functionalized region on the SIW, air holes were introduced on the strongest E-field. Without using any additional coating layers, sensitivity was enhanced owing to a larger functionalized area. An SIW resonator working in transverse-electric (TE) modes specifically TE_101_ and TE_102_ was proposed as a gas detection sensor [8]. Tin oxide (SnO_2_) powder, being reactive toward hydrogen gas (analyte), was poured to functionalize a specific area (encompassing the strongest E-field). To investigate the effect of material-dependent properties on the sensitivity, several analyses were conducted, such as dimensions of the functionalized area, substrate permittivity, and grain size of the SnO_2_ powder on the sensitivity of the device. The transverse-electric (TE) modes of rectangular metallic waveguides also exist in SIWs because the propagation characteristics of the SIW are similar to those of the metallic waveguide, provided that the metallic vias of the SIW are closely spaced and the radiation leakage is neglected [9]. In the following paragraph, the evolution of the quarter-mode substrate-integrated waveguide (QMSIW) is detailed. 

Several attempts have been made to reduce the size of SIWs while maintaining or even enhancing the performance. Various RF applications have been realized, such as half-mode SIWs (HMSIWs) [10,11], quarter-mode SIWs (QMSIWs) [12,13], eight-mode SIWs (EMSIWs) [14,15], sixteen-mode SIWs [16], and ridged quarter-mode SIWs [17]. According to image theory, in-phase fields exist on opposite sides; thus, the symmetric plane of an SIW is cut into two halves to obtain an HMSIW [12]. The HMSIW has almost the same E-field distribution as the full SIW, while its size is halved. Owing to the fringing fields on the edges of the HMSIW, the equivalent size of the HMSIW is smaller than the half of that of the corresponding full SIW [18]. Further bisection of an HMSIW is performed to obtain the quarter-mode SIW (QMSIW). In a QMSIW, the E fields are further reduced to some extent; however, the QMSIW is adequate for sensing and tuning applications [17,18,19]. Being a quadrant of an SIW, the size of a QMSIW is one-fourth that of a full SIW. In [18], a QMSIW was excited in the TE_101_ and TE_202_ modes to achieve linearly polarized and circularly polarized radiation, respectively. In [19], a triple-mode filter was realized using an isosceles right-angle triangular waveguide with consideration of two open edges as magnetic walls and one electric wall along the hypotenuse. In addition to TE modes, transverse-magnetic (TM) modes are supported in a triangular QMSIW structure [19]. In [20], a novel flow sensor was proposed to measure the fluidic flow. A microfluidic channel covered with a polydimethylsiloxane (PDMS)-based membrane served as the microfluidic device which was integrated with a half-wavelength microwave resonator. The highest sensitivity recorded with this noncontact and nonintrusive flow sensor was 0.5 µL/min for a thin membrane which was 3 mm in diameter and 100 µm thick.

Metamaterial (MM) unit cells are arranged to realize periodic structures [21,22] that simultaneously exhibit negative permittivity and negative permeability [23,24,25]. MM-inspired structures provide a higher quality factor (Q) and are smaller than SIW-based cavity resonators, half-wavelength resonators, and so forth. The geometry, topology, and way in which they are configured, using either an electric or a magnetic field, determine their response and operating-frequency range [26,27]. MM topologies, for example, split-ring resonators (SRRs) and complementary SRRs, are widely utilized to realize diverse RF components [28,29]. Recently, SRR-based chemical sensors have been proposed for detecting two or more chemicals. For instance, in [30], four unique resonance frequencies were tuned using 5 µL of ethanol in an experimental demonstration. In [31], a microfluidic dual-channel resonator consisting of three open-SRRs with unequal dimensions was reported. The independent tuning of one resonance was demonstrated. In [32], two SRRs coupled with a microstrip line were proposed as a dual-detection chemical sensor. This sensor is based on the frequency-splitting phenomenon and requires two asymmetric dielectric chemicals to be loaded. If the same dielectric chemical was loaded in both channels, a frequency shift was not observed. In [33], a dual-mode resonator was proposed as a dual sensor. In one mode, the resonance-shift principle is based on the capacitive effect, while in the other mode, the properties of a stub (i.e., the change of the characteristic impedance and the electrical length) are used. In Section 5, we compare the performance of our proposed sensor and the aforementioned dual/multiple detection RF sensors.

The TM_02_-mode QMSIW design used in this study is inspired by [18,19], and we applied it to extend our previous work [34], in which a TE_20_-mode full SIW was proposed as a dual-detection microwave chemical sensor. The main contribution of the present study is miniaturization together with simultaneous dual detection using a microwave sensor. The size is reduced mainly by utilizing a QMSIW resonator and a substrate with a high dielectric constant. Unlike our previous dual-detection microwave sensor [34], in which polydimethylsiloxane (PDMS) was inserted between two main substrates to realize a sandwich-like structure, only a single main substrate (Rogers RT/duroid) is utilized in the present study. The details and analysis are presented in Section 2. 

Herein, a TM_02_-mode QMSIW resonator that can simultaneously detect two analytes using a single-chip sensor is proposed. Two asymmetric microfluidic channels have been designed to be loaded on two distinct E-field regions to perturb the effective permittivity of the localized regions in the substrate. All possible combinations of ethanol and deionized (DI) water are loaded in the channels, and the distinct resonance frequency in each case is reported. The design guidelines, fabrication, and measurements are illustrated. In addition, the salient features of the proposed sensor are compared with those of existing multiple-detection sensors.

## 2. Sensor Design

### 2.1. Theory

**Figure 1:**
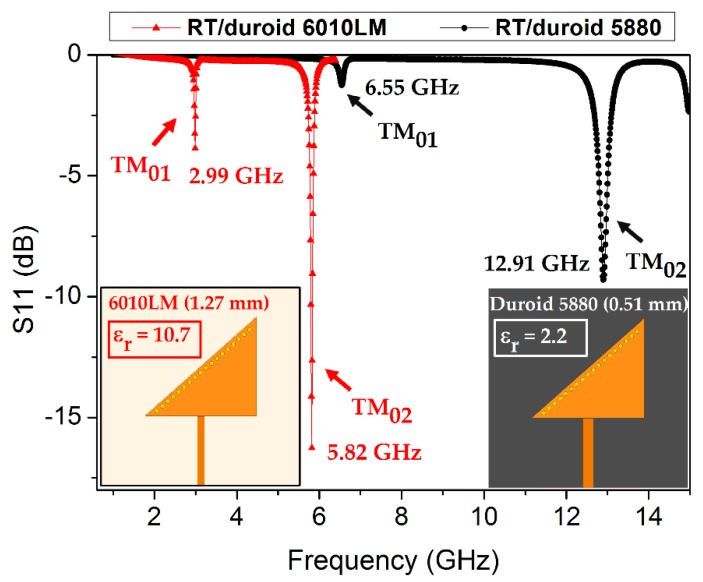
TM_02_-mode QMSIW resonator realized on two substrates having significantly different dielectric constants; the corresponding return loss (S11) and resonance frequencies are shown. RT/duroid 6010LM (ε_r_ = 10.7) exhibits a significant downshift in the resonance frequency, which ensures a compact design compared with the design realized on RT/duroid 5880 (ε_r_ = 2.2). The TM_02_-mode in each case is verified after analyzing the E-field magnitude distribution, which is not shown here for brevity.

The dielectric constant (relative permittivity), which indicates an ability associated with dielectric materials, tends to measure how easy or difficult it is to polarize a material upon excitation of the external E-field and the consequent storage of energy. Rogers Corporation Inc. (Chandler, AZ, USA) provides specially designed microwave low-loss substrates with highly stable dielectric constants, such as 5880, 5870, 3010, and 6010LM [35,36]. Substrate materials having low dielectric constants (ε_r_) have been extensively utilized in various RF designs. The choice of the feeding technique, design approach/technology, and substrate material plays a key role in the performance and size of the design. Substrates having high dielectric constants (ε_r_ ≈ 10) are known to facilitate miniaturization [37]. To find a suitable substrate, a design with the same dimensions of the triangular patch are realized on two different substrates: namely, RT/duroid 5880 (ε_r_ = 2.2, thickness 0.51 mm) and RT/duroid 6010LM (ε_r_ = 10.7, thickness h_s_ =1.27 mm). The resonance frequency is observed at 12.91 and 5.82 GHz, and the results for the return loss obtained from these simulations are shown in Figure 1. The 6010LM substrate is an obvious choice for developing a compact TM_02_-mode QMSIW resonator without compromising on performance; therefore, it is chosen as the main substrate in this study.

If the height (thickness of substrate used, in our case h_s_ = 1.27 mm) of an SIW cavity resonator is far smaller than its length (*L_SIW_*) and width (*W_SIW_*), the resonance frequency of the resonator can be defined as

(1) $$f_{mn0}=\frac{1}{2\pi \sqrt{\mu \varepsilon }}\sqrt{{\left(\frac{m\pi }{W_{SIW}}\right)}^{2}+{\left(\frac{n\pi }{L_{SIW}}\right)}^{2}}$$

where *µ* and *ε* represent the permittivity and permeability, respectively, of the dielectric material and *m* and *n* are the integer-mode indices. According to Equation (1), TE_100_ is the lowest resonance frequency [13] and TE_101_ is the dominant resonant mode [38]. The desired resonant frequency can be tuned by adjusting the length and width of the SIW cavity resonator and the effective permittivity of the dielectric material. Because a QMSIW maintains/preserves almost the same E-field distribution as an SIW, Equation (1) can be applied to determine the resonance frequency in a specific mode of a QMSIW.

(2) $$f_{02}=f_{20}=\frac{1}{2\pi \sqrt{\mu \varepsilon }}\sqrt{{\left(\frac{2\pi }{W_{QMSIW}}\right)}^{2}}$$

For a TM_02_-mode QMSIW resonator, Equation (2) is modified to include the fringing effects arising from the open-ended edges of the triangular QMSIW:

(3) $$f_{02}=\frac{1}{2\pi \sqrt{\mu \varepsilon }}\frac{2\pi }{W_{QMSIW}+\Delta W}$$

where *W_QMSIW_* is the physical width of the QMSIW patch and Δ*W* is the extended width due to fringing fields. Therefore, the dimensions of this QMSIW cavity resonator are smaller than those of a quarter of an SIW.

### 2.2. Design of Dual-Detection Chemical Sensor

**Figure 2:**
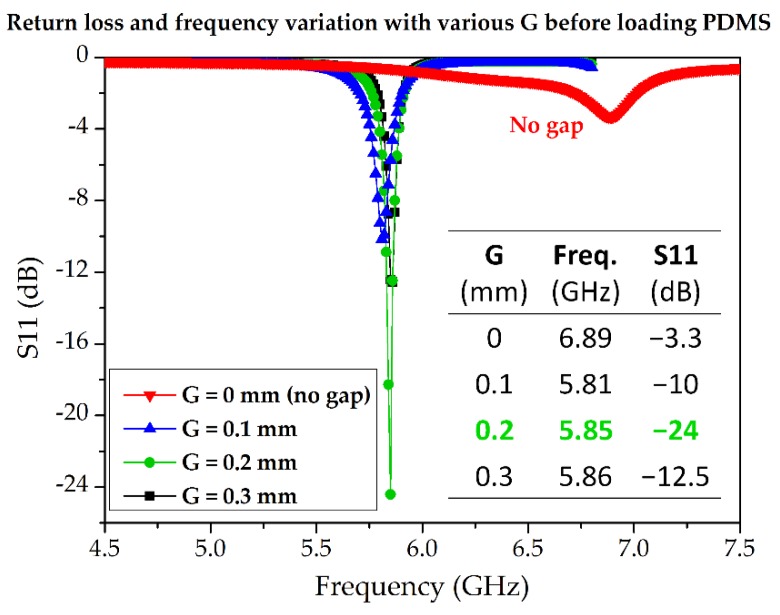
Return loss and notch frequency variation for various coupling gaps (G) between the microstrip line and the TM_02_-mode QMSIW triangular patch. The coupling gap of *G* = 0.2 mm yields the best impedance matching before the loading of the microfluidic channels. The largest notch frequency variation is 40 MHz while G changes from 0.1 mm to 0.2 mm.

In this subsection, we describe the design process for the TM_02_-mode triangular-patch QMSIW. The conductive QMSIW triangular patch and ground are realized on the top and bottom of a Rogers RT/duroid 6010LM substrate (ε_r_ = 10.7, tan Δ = 0.0023, h_s_ = 1.27 mm). The dimensions of the substrate are *L_s_* × *W_s_* = 30 mm × 30 mm. The dimensions of the right-triangular QMSIW resonator are chosen as *W × L* = 17 mm × 15 mm to resonate at 5.81 GHz. There is no reason to choose this particular frequency except that testing and comparison with state-of-the-art microwave sensors show it to be reasonable. Metallic vias (copper) are infused along the hypotenuse of the triangular patch to connect the top and bottom grounds for realizing an electric wall along the hypotenuse and two magnetic sidewalls along the open-ended edges of the triangular QMSIW resonator. Consequently, the E-field remains preserved inside the triangular patch, which can be used for sensing. The performance of the QMSIW is improved by minimizing the leakage/radiation losses, such that *D* < *λ_g_*/5 and *P* < 2*D*, where *D, P*, and *λ_g_* are the via diameter, the pitch, and the guided wavelength, respectively [39,40]. The values of *D* and *P* are determined to be 0.6 and 1.1 mm, respectively. A 50 Ω microstrip line is commonly used to excite the microwave resonators as a preferred feeding technique. However, once the dielectric constant and thickness of the substrate are chosen, the width of the microstrip line is almost fixed, with a narrow margin of variation. The dimensions (length and width) of the microstrip line are *L_m_* × *W_m_* = 10.5 mm × 1.1 mm. The microstrip line (50 Ω) coupled with the triangular patch realized on 6010LM failed to produce reasonable impedance matching. Alternatively, the coupling-gap feeding technique is considered. In order to find the required coupling, various gaps (*G*) between the triangular patch and the microstrip line are investigated, as shown in Figure 2. Three cases of coupling gap (G = 0.1 mm, 0.2 mm, and 0.3 mm) are considered. Variation in notch frequency of 40 MHz and 10 MHz are observed when G changes from 0. 1 mm to 0.2 mm and then from 0.2 mm to 0.3 mm, respectively. *G* = 0.2 mm is determined to yield critical coupling; however, after the loading of the channels (discussed in the next subsection), the optimum coupling gap should be re-investigated.

**Figure 3:**
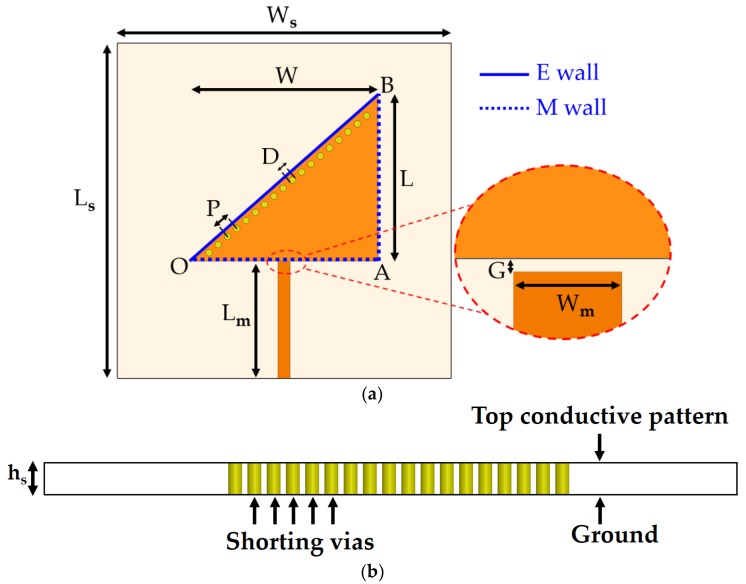
Geometry of the TM_02_-mode QMSIW resonator proposed as a dual-detection chemical sensor: (**a**) top view. L_s_ and W_s_ represent substrate’s (length and width), L_m_ and W_m_ represent microstrip line’s (length and width), OA, AB and OB represent dimensions of triangular patch; (**b**) cross-sectional view.

The physical size of the proposed TM_02_-mode QMSIW resonator, including the microstrip line, is 25.6 mm × 17 mm. Its electrical size is 1.62*λ_g_* × 1.07*λ_g_* at 5.81 GHz. The proposed TM_02_-mode QMSIW resonator—without the loading of microfluidic channels—is shown in Figure 3.

### 2.3. Design of Asymmetric Microfluidic Channels

**Figure 4:**
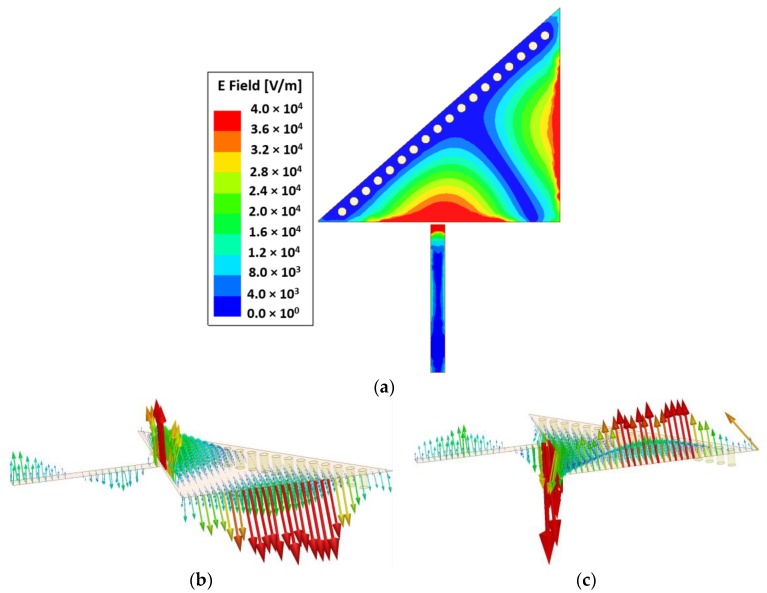
(**a**) E-field magnitude of the proposed TM_02_-mode QMSIW resonator at the resonance frequency of 5.81 GHz. Two distinct strong E-field regions are the best place to load two microfluidic channels. E-field vectors (vertical distribution) of the proposed TM_02_-mode QMSIW resonator during (**b**) a positive half cycle and (**c**) a negative half cycle are shown. There is a clear distinction between the two strong E-field regions, as shown in (**a**,**b**). This ensures the realization of two closely spaced yet noninterfering channels.

To change the effective permittivity of the dielectric material, microfluidic channels are loaded in the strongest E-field regions; consequently, a higher sensitivity can be expected. The E-field magnitude of the TM_02_-mode QMSIW resonator is shown in Figure 4. Two distinct E-field regions can be manipulated using two microfluidic channels. In our previous study, two symmetric channels of equal fluid-carrying capacity were unable to produce distinct resonance frequencies when loaded with [E, DI water] and [DI water, E] [34]. Therefore, two channels having unequal fluid-carrying capacities must be realized.

**Figure 5:**
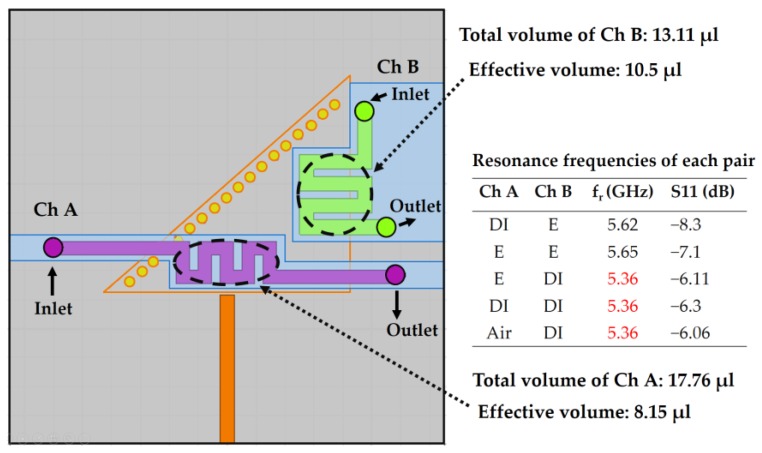
Simulation of two asymmetric channels, unable to produce a unique resonant frequency in each case, which can be explained by reasoning that the effective volumes (equivalent fluidic volume that overlaps with the strongest E-field) of the channels differ by a narrow margin. Although the channels are intentionally designed to be asymmetric, the effective permittivity is perturbed in a uniform way owing to the inadequate difference between the effective volume of channels: Ch A and Ch B. Therefore, different combinations of channel loadings—such as [E, DI], [DI, DI], and [Air, DI]—are observed to generate exactly the same resonance frequency. E and DI represent ethanol and deionized water, respectively.

To investigate the influence of the volume in the dual-detection process, two meander-shaped asymmetric channels (Ch A and Ch B) are designed, as shown in Figure 5. Although their fluid-carrying capacities are unequal—the volume of Ch A = 17.76 µL and the volume of Ch B = 13.11 µL—the resonance frequency in each channel-loading case was not unique. According to the dimensions, the effective volumes of Ch A and Ch B are estimated as 8.15 and 10.50 µL, respectively. It is expected that the difference between the effective volumes of these channels is insufficient to generate a unique resonant frequency in each case.

### 2.4. Optimized Geometry and Effective Volume of Microfluidic Channels

If the thickness of both channels is kept constant and the overlapped area of one channel differs significantly from that of the other channel, different resonance frequencies in each case can be expected. The overlapped area represents the only part of the channel that covers the E-field on the QMSIW patch, ignoring the part of the channel that covers the 6010LM substrate owing to its noninfluential contribution. To overlap the maximum area of the strongest E-field, the channels are designed as inverted-U-shaped (Ch 1) and meander-shaped (Ch 2). The length of the PDMS sheet containing Ch 1 and the width of the PDMS sheet containing Ch 2 are designed to eliminate the need for alignment marks. Both channels—each having a depth of h_c_ = 0.6 mm—are engraved in a PDMS layer (h_p_ = 1mm). A double-sided adhesive bonding film (ε_r_ = 3.4, tan Δ = 0.03, and h_f_ = 0.05 mm) is considered below for the PDMS-based channels to ensure the noncontact feature of the microwave sensor as well as to maintain the position of the PDMS.

**Figure 6:**
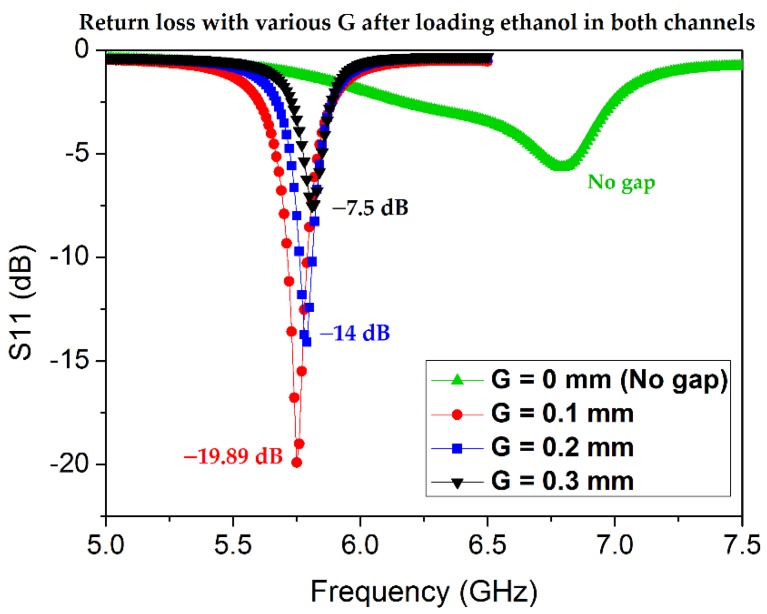
Parametric analysis for investigating the critical coupling gap for the final design. *G* = 0.1 mm is found to be the best impedance-matched case under the criterion that the microfluidic channels are loaded with ethanol.

After the microfluidic channels are loaded with the lossy liquids, the impedance matching is disrupted and the relevant design parameters (e.g., the coupling gap *G*) must be redesigned. Because ethanol is a lossy liquid with a high loss tangent, instead of empty channels, both channels filled with ethanol are subjected to impedance matching. A parametric analysis is conducted to determine the optimum coupling gap after the microfluidic channels are loaded with ethanol, as shown in Figure 6. The return-loss values are enhanced for the ethanol-filled channels. Before the loading of the channels, the electromagnetic (EM) waves pass through air (ε_r_ = 1), and the same EM waves pass through the ethanol-filled PDMS-based channel; thus, the impedance matching is improved (ε_r_ of ethanol > ε_r_ of PDMS > ε_r_ of air). *G* = 0.1 mm is found to be the best impedance-matched case and is used for the final design.

**Figure 7:**
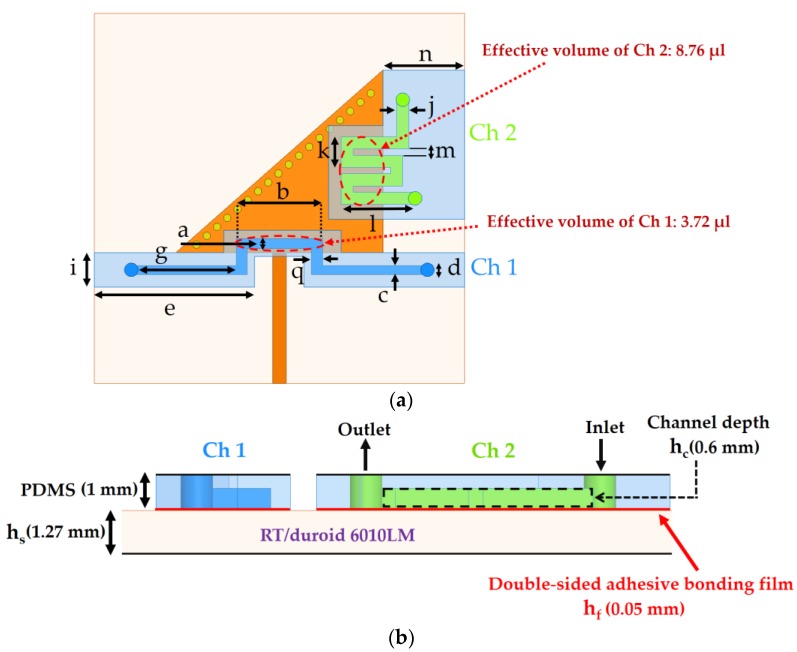
Layout of the proposed TM_02_-mode triangular QMSIW resonator as a dual-detection chemical sensor: (**a**) top view; (**b**) cross-sectional side view. 1 mm-PDMS sheet is considered to model microfluidic channels (h_c_ = 0.6 mm) and an adhesive film (h_f_ = 0.05 mm) is used for bonding purpose. The effective volumes of the two channels are estimated and found to differ considerably, as shown in (**a**).

The final layout of the proposed TM_02_-mode QMSIW resonator with microfluidic channels is shown in Figure 7.

According to the channel dimensions (see Figure 7), the fluid-carrying capacities (total volume) are estimated as 11.21 and 14.34 µL for Ch 1 and Ch 2, respectively. The parameters for our final design are listed in Table 1.

## 3. Simulation Analysis

**Figure 8:**
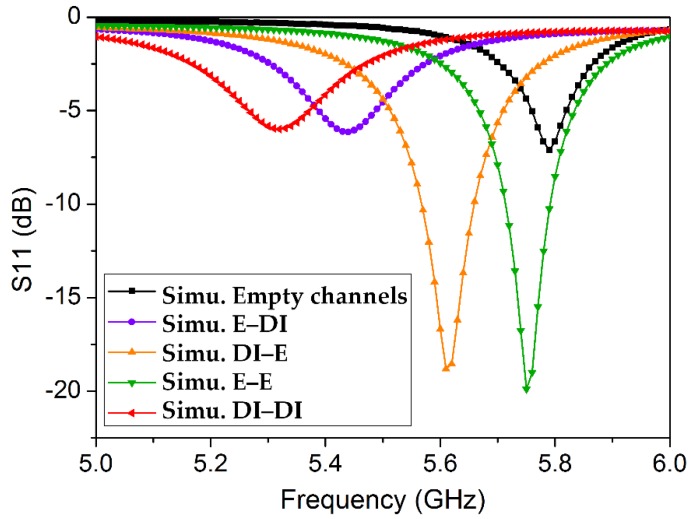
Return loss (simulated) for empty channels and four possible combinations of ethanol (E) and DI water (DI) in the channels, loaded onto the TM_02_-mode QMSIW resonator.

To analyze the dual-detection capability of the proposed TM_02_-mode QMSIW resonator as a microwave sensor, microfluidic channels are characterized using the dielectric properties of empty channels and four combinations of ethanol (E) and/or DI water (DI). The simulated return-loss results are shown in Figure 8. The dielectric properties of the 1-mm PDMS layer were assumed to be ε_r_ = 2.7 and tan Δ = 0.05 [41]. The structure resonates at 5.79 GHz when both channels are empty. Full-wave simulations are conducted using a high-frequency structure simulator, and four different resonance frequencies of 5.44, 5.61, 5.75, and 5.32 GHz are obtained, corresponding to [E, DI], [DI, E], [E, E], and [DI, DI], respectively. The dielectric properties of ethanol and DI water are set as ε_r_ = 5.08 and tan Δ = 0.4 [42] and ε_r_ = 73 and tan Δ = 0.3 [43], respectively. After the microfluidic channels were realized, the unloaded quality factor Q (simulated) was calculated as Q ≈ 43 using a well-known formula [44,45].

### Sensitivity Analysis

**Figure 9:**
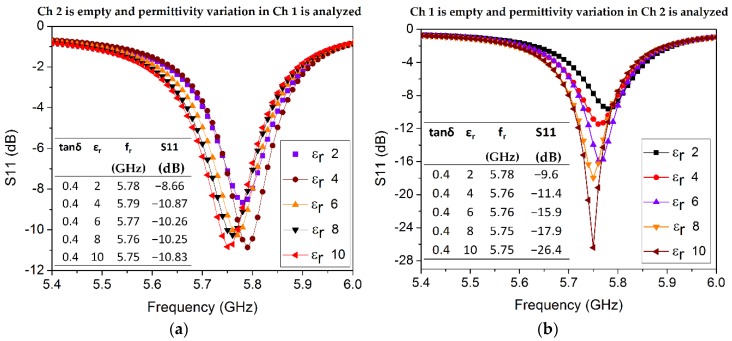
Effect of permittivity variation of material inside the channel on the sensitivity of the proposed sensor. (**a**) Ch 2 is empty, and permittivity variation in Ch 1 is analyzed. (**b**) Ch 1 is empty, and permittivity variation in Ch 2 is analyzed. Loss tangent value is considered as a constant (tan δ = 0.4) during these sets of simulations.

In order to investigate the sensitivity of both channels, a parametric analysis is conducted to observe variation in the dielectric properties of the channel material. First, the permittivity variation in each channel is individually investigated for ε_r_ values ranging from 2 to 10 with a step size of 2 (see Figure 9). Loss tangent (tan δ) is considered as a fixed parameter in both sets of simulations and is arbitrarily chosen as 0.4, which is a common value of the loss tangent of ethanol around 3–5 GHz. The dielectric properties of the empty channel are set as ε_r_ = 1 and tan δ = 0 in simulations. Δf and Δε are calculated from each pair of resonant frequency and corresponding permittivity values, provided in the inset tables in Figure 9. For instance, S1 represents the average sensitivity of Ch 1 upon permittivity variation and is estimated as follows:

$$S1=\frac{\Delta f}{\Delta \varepsilon }=\frac{{\Delta f}_{1}{+\Delta f}_{2}{+\Delta f}_{3}{+\Delta f}_{4}}{{\Delta \varepsilon }_{1}{+\Delta \varepsilon }_{2}{+\Delta \varepsilon }_{3}{+\Delta \varepsilon }_{4}}=\frac{10+20+0+10}{2+2+2+2}=6.25\mathrm{MHz}/\varepsilon_{r}$$

With the data taken from Figure 9b and applying the above equation, the average sensitivity of Ch 2 upon permittivity variation is calculated as S2 = 2.5 MHz/ε_r_. A higher sensitivity of Ch 1 is perhaps due to more overlapped area containing a stronger E-field.

**Figure 10:**
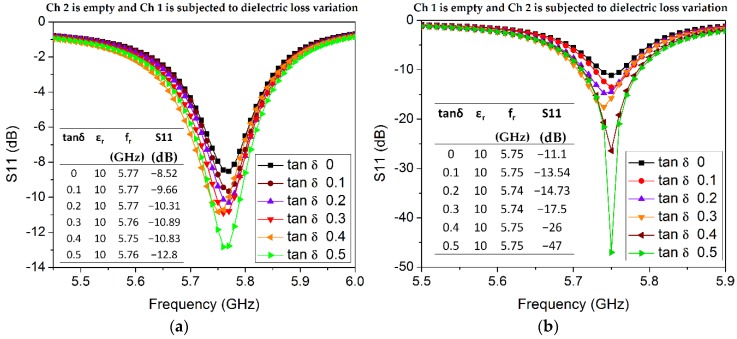
Effect of loss tangent variation of material inside the channel on the sensitivity of the proposed sensor. (**a**) Ch 2 is empty, and loss tangent variation in Ch 1 is analyzed. (**b**) Ch 1 is empty, and loss tangent variation in Ch 2 is analyzed. Permittivity is considered as a constant (ε_r_ = 10) during these sets of simulations.

Now, the influence of the loss tangent inside each channel (material) on the sensitivity of our proposed sensor is investigated (see Figure 10). Loss tangent (tan δ from 0 to 0.5 with an incremental step of 0.1) and a fixed permittivity value (arbitrarily chosen as ε_r_ = 10) are assigned to Ch 1 while the Ch 2 is considered as empty, and vice versa. As we know, the loss tangent is associated with the return loss magnitude; for instance, the return loss of Ch 1 varies from 8.5 dB to 12.8 dB when tan δ changes from 0 to 0.5, while the resonant frequency almost remains constant with a slight shift of 10 MHz (see inset table provided in Figure 10a). From Figure 10b, the return loss changes from 11 dB to 47 dB when tan δ varies from 0 to 0.5. An equal amount of incremental change in tan δ causes a larger variation in the return loss (magnitude) of Ch 2 as compared with Ch 1, which can be explained by reasoning that the volume of Ch 2 is higher than that of Ch 1.

## 4. Fabrication and Measurement

### 4.1. Fabrication

**Figure 11:**
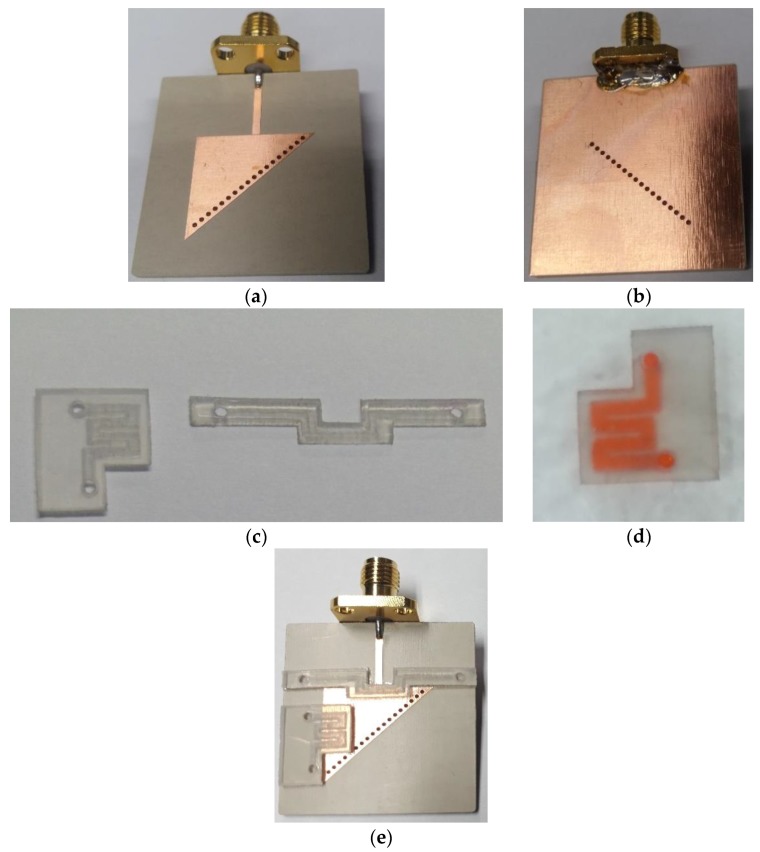
(**a**) Fabricated prototype integrated with microfluidic channels. (**a**,**b**) Top/bottom views of the fabricated prototype. (**c**) Both the channels realized using a laser cutting machine. (**d**) Testing of the channels by injecting colored water for verification (only Ch 2 is shown). (**e**) Fabricated prototype resonator integrated with microfluidic channels.

The conductive pattern and ground are realized on the top and bottom of a Rogers RT/duroid 6010LM substrate using conventional photolithography. Unlike our previous dual-detection microwave sensor, this study is based on a single substrate integrated with two asymmetric microfluidic channels loaded in two E-field regions. The two PDMS-based microfluidic channels were fabricated using a laser cutting machine, which was a quick process, although the channel surfaces are slightly rough. The fabricated prototype resonator, the channels engraved inside the PDMS slabs, and the demonstration of the fluid-carrying capability are presented in Figure 11. An adhesive film is used to bond the PDMS-based channels onto the conductive pattern, which is realized on duroid 6010LM, as shown in Figure 11e.

### 4.2. Measurement

**Figure 12:**
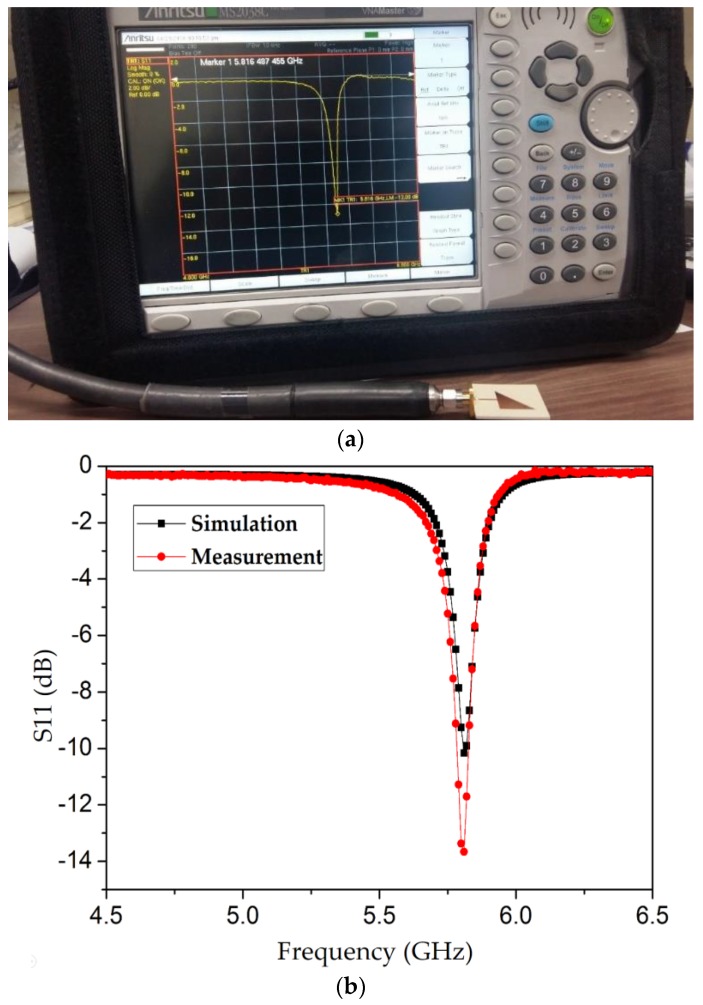
(**a**) Measurement setup: the proposed TM_02_-mode triangular QMSIW resonator (without loading of microfluidic channels) is measured using a vector network analyzer and resonates at 5.81 GHz. (**b**) Simulated and measured return-loss values obtained for the proposed resonator without the loading of microfluidic channels. The simulated unloaded Q ≈ 43 is calculated according to a 3-dB bandwidth at the resonance frequency.

The fabricated prototype integrated with microfluidic channels is connected to a vector network analyzer (Anritsu MS2038C, manufactured by Anritsu Corporation Kanagawa Prefecture, Japan). The measurement setup is shown in Figure 12a. The return loss is measured when the channels are empty and compared with the simulation results, as shown in Figure 12b. The simulated and measured resonance frequencies (5.81 GHz) exhibit excellent agreement. 

**Figure 13:**
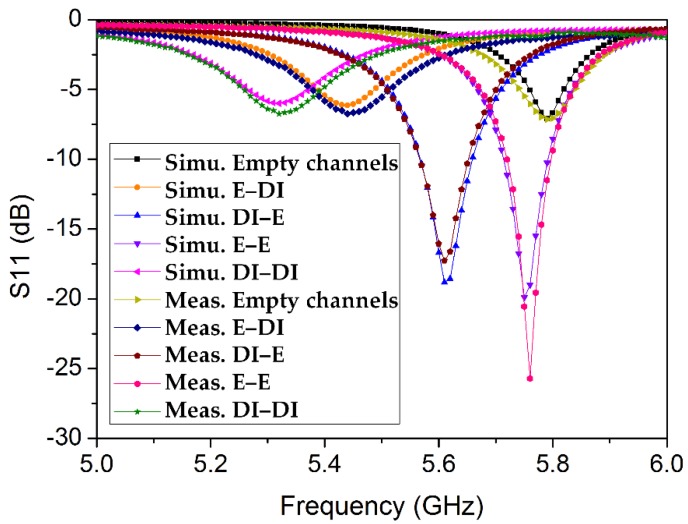
Comparison of the return-loss values obtained from the simulation and measurement for all possible combinations of ethanol and DI water and for empty channels.

To demonstrate the dual-detection capability, ethanol and DI water are alternately injected into the channels, in four possible combinations (see Figure 13). The resonance frequency and return loss in each case obtained from the simulation and measurement are compared in Table 2. To evaluate the accuracy of the measurement, the relative error in the resonance frequency is calculated (referring to Equation (3) from [34]).

## 5. Discussion

To evaluate the proposed microwave resonator, its performance (frequency shifts and electrical size) is compared with that of recently published SIW resonators, as shown in Table 3. Our microwave resonator exhibits comparable performance; moreover, the size is reduced. Notably, the applications of the other SIW resonators in Table 3 are limited to a single analyte.

The performance of our dual-detection chemical sensor was compared with that of existing microwave dual/multiple detection sensors, as shown in Table 4. To evaluate the performance of these sensors, the fractional frequency shift was calculated, which is defined as $S=\frac{\Delta f}{f_{o}}\times 100$, where Δ*f* represents the absolute frequency shift and *f_o_* is the operating frequency. The unit of the sensitivity *S* is (%). A qualitative analysis of our proposed sensor compared with existing multichannel sensors is provided in Table 5.

Misalignment of the channels in the fabricated prototype may lead to inaccurate measurements and a compromised sensitivity [47]. The authors consider that such misalignment cannot be eradicated via manual handling; however, it can be avoided to some extent using alignment marks on the sensor chip. To investigate this, the dimensions of PDMS slabs (containing channels) were designed to cause overlap along the width of substrate or along one side of the QMSIW patch (as discussed in detail in Section 2).

As indicated by Table 3, single-analyte sensors may suffer in certain scenarios, as discussed in our previous work [34]. A dual/multiple sensor similar to that proposed herein can provide reliable detection in the case where one channel is biased [48,49].

In [30], a microwave resonator capable of detecting four different chemicals is presented; however, it is a direct-contact sensor, which may raise concerns such as contamination, as discussed in [34]. These issues are prevented in our proposed microfluidic integrated microwave resonator.

Among the dual/multiple detection microwave sensors compared in Table 4, only those from [30,33] exhibit independent tuning capability. The tuning of the resonance frequencies in our proposed sensor is partially independently-tunable, owing to its inherent nature (two E-field regions tend to control a single resonance). The sensor proposed in [33] is comprised of five stacked layers constructed using low-temperature cofired ceramic technology and laser micromachining.

RF/microwave chemical sensors suffer from being nonselective. To resolve this issue, hybrid sensors (RF sensor with additional coatings) have been proposed [49,50].

Every sensor is designed to meet certain performance criteria or optimized to target a specific application. Our proposed sensor has a simple design and is fabricated using conventional lithography; thus, it is inexpensive. Being noncontact and having sensitivity comparable to that of contemporary microwave sensors, the TM_02_-mode QMSIW resonator has potential as a dual-detection microwave sensor.

## 6. Conclusions

A TM_02_-mode triangular QMSIW resonator is proposed for the detection of two chemicals regardless of their dielectric constants. The novelty of the proposed resonator is its dual-detection capability, and size reduction is achieved using the QMSIW approach. Loading two asymmetric channels in two distinct E-field regions was insufficient to obtain different resonance frequencies. A certain difference in the effective volume of each channel must exist in order to obtain the unique resonance regardless of the dielectric constants of the channel loadings. To demonstrate the potential of the proposed dual-detection chemical sensor, ethanol and DI water were injected into the microfluidic channels, and return-loss measurements agreed well with simulations. The performance of the proposed sensor can be improved with regard to the sensitivity and size reduction. To improve the sensitivity of the dual-detection sensor, the overlapped area of both channels can be increased, for example, by using closely spaced meander-shaped channels. Further miniaturization can be achieved, for example, by using a sixteen-mode SIW resonator.

## Figures and Tables

**Table 1:** Design parameters for the TM_20_-mode QMSIW resonator (unit: mm).

Parameter	Value	Parameter	Value	Parameter	Value
L_s_	30	G	0.1	i	2.8
W_s_	30	h_s_	1.27	j	1
L	15	a	0.8	k	2.5
W	17	b	7	l	5.5
D	0.6	c	0.7	m	0.5
P	1.1	d	1.1	q	0.9
L_m_	10.5	e	13	h_f_	0.05
W_m_	1.1	g	8	h_c_	0.6

Note that parameters are already defined according to Figure 7 and the Section 2.

**Table 2:** Comparison between the simulation and measurement results after the loading of microfluidic channels. The resonant frequencies and return-loss measurements are provided, corresponding to empty channels and the four possible combinations of ethanol and DI water.

Ch 1, Ch 2	Simu. f_r_ (GHz)	Simu. S11 (dB)	Meas. f_r_ (GHz)	Meas. S11 (dB)	Relative Error in f_r_ (%)
Air, Air	5.79	−7.10	5.791	−7.14	0.02
Ethanol, DI water	5.44	−6.14	5.445	−6.74	0.09
DI water, Ethanol	5.61	−18.81	5.614	−17.28	0.07
Ethanol, Ethanol	5.75	−19.89	5.760	−25.71	0.17
DI water, DI water	5.32	−5.99	5.321	−6.74	0.02

**Table 3:** Comparison of our TM_02_-mode QMSIW resonator with recently proposed SIW chemical sensors.

Ref.	f_o_ (GHz)	Δf_max_ * (MHz)	Technology	Electrical Length λ_g_ × λ_g_	Sensing
This work	5.81	470	QMSIW	1.62 × 1.07	Dual
[14]	4.65	400	EMSIW	0.94 × 0.9	Single
[40]	17.08	610	SIW	3 × 2.61	Single
[44]	5	380	SIW	1.85 × 1.85	Single
[46]	13.48	170	SIW	2.26 × 1.92	Single

* Δf_max_ represents the maximum frequency shift (absolute value) of the device among all the test cases.

**Table 4:** Performance comparison between our proposed TM_02_-mode QMSIW resonator and other microwave dual/multichannel sensors.

Ref.	f_o_ (GHz)	Δf * (MHz)	S (%)	Physical Size (mm × mm)	Electrical Size (λ_g_ × λ_g_)
[34]	8	430	5.37	60 × 40	2.37 × 1.58
[30]	3	170	5.66	35 × 32	1.12 × 1.02
[31]	6.5	400	6.15	30 × 22	1.13 × 0.86
[32]	0.87	110	12.6	86 × 62	0.8 × 0.57
[33]	2	200	10	50 × 40	1.4 × 1.12
This work	5.77	31	8.1	30 × 30	1.62 × 1.07

* Δf represents the absolute frequency shift when frequencies corresponding to ethanol/DI water are compared with that for the empty channel. Conventionally, frequency shifts in RF chemical sensors are compared with reference to DI water. However, because of the insufficient data in some reports, air was used as the reference media. Some studies, such as [30], demonstrated multichannel sensing without the utilization of microfluidic channels.

**Table 5:** Qualitative features of our proposed TM_02_-mode QMSIW resonator along with other microwave chemical sensors capable of dual/multiple detection.

Ref.	Technology	Configuration	Noncontact	Independent Tuning	Detection
This work	TM_02_-mode QMSIW	Unit cell on single substrate	Yes	No	Dual
[34]	TE_20_-mode SIW	Stacked layer	Yes	No	Dual
[30]	MM	Array	No	Yes	Multiple
[31]	MM	Array	Yes	Yes	Dual
[32]	MM	Array	Yes	No	Partially Dual
[33]	Dual-mode resonator	Stacked layer	Yes	Yes	Dual

Note: Topologies in [30,31,32] are 4 SRRs, 3 open split ring resonators (OSRRs), and 2 SRRs, respectively, whereas MM represents metamaterial technology.
